# Influence of Phospholipid Composition on Protein Adsorption to Lipid-Coated Silica Microparticles

**DOI:** 10.3390/molecules31060966

**Published:** 2026-03-13

**Authors:** Mireia Vilar-Hernández, Dorothee Wasserberg, Jasper van Weerd, Pascal Jonkheijm

**Affiliations:** 1 Laboratory of Biointerface Chemistry, Department of Molecules and Materials, University of Twente, P.O. Box 217, 7500 AE Enschede, The Netherlands; m.vilarihernandez@utwente.nl (M.V.-H.);; 2 LipoCoat BV, Hengelosestraat 541, 7521 AG Enschede, The Netherlands

**Keywords:** silica particle, phospholipid coating, lipid bilayer, protein corona

## Abstract

Silica particles are promising multifunctional drug delivery platforms; however, when in contact with blood or other biological fluids, proteins rapidly adsorb to their surface, forming the protein corona that modulates their biological interactions. In this study, silica microparticles were coated with lipid bilayers using two approaches: the lipid film hydration method and the on-particle solvent-assisted lipid coating (OPSALC) technique. We investigated how phospholipids with varying charges (zwitterionic, anionic, and cationic) and membrane phase influence coating formation and protein corona adsorption. The coating coverage and aggregation were characterized by fluorescence microscopy. The lipid film hydration method enabled coating with a broad range of lipids, but was highly dependent on the membrane phase and electrostatic interactions between lipid head group and particle surface. Pure anionic coatings were not achievable with this method; however, when combining the OPSALC method with a pre-silanization step, fully anionic coatings of silica microparticles were successfully obtained. Assessment by SDS-PAGE revealed differences in protein corona profiles modulated by the lipid compositions on the particles’ coatings. Overall, this study highlights the dependence of coating formation and protein corona composition on the phospholipid coatings’ properties.

## 1. Introduction

Silica and porous silica particles have attracted considerable interest as multifunctional drug delivery platforms owing to their tunable physicochemical properties that address challenges such as poor solubility, limited biodistribution, and insufficient target specificity [1]. Because microparticles are generally not ideal for extended circulation in the bloodstream, instead they are commonly employed for localized, sustained release of therapeutics through subcutaneous, intramuscular, or intraperitoneal injections, rather than intravenous administration [2,3,4]. However, when particles come into contact with biological fluids such as blood plasma, proteins rapidly adsorb to their surface, forming an adsorbed protein layer often referred to as a protein corona, a term widely used in nanoparticle literature [5]. This protein corona is strongly influenced by the physicochemical properties of the particles, such as size, morphology, charge, surface chemistry, and hydrophobicity [6,7,8,9]. The protein corona often includes immunoglobulins and complement proteins that act as opsonins, promoting recognition by phagocytic cells through Fc and complement receptors [10,11]. The resulting opsonization facilitates phagocytosis and rapid clearance by the mononuclear phagocyte system, particularly in the liver, spleen, and lungs [12,13]. To minimize opsonization and prolong residence time, microparticles can be coated with hydrophilic surface layers, such as polyethylene glycol (PEG). Although PEG remains the gold standard for non-fouling coatings, concerns over oxidation and the emergence of anti-PEG antibodies have prompted growing interest in alternative non-fouling coatings [14,15,16,17]. Alternatives include lipid-based coatings that form supported lipid bilayers on particle surfaces [18]. Such coatings mimic key structural and physicochemical features of biological membranes, including zwitterionic phospholipid headgroups, a highly hydrated interface, and lateral lipid mobility [19]. These characteristics can reduce nonspecific protein adsorption and influence the composition of the protein corona, thereby modulating immune recognition and particle–biointerface interactions [18,19]. For this reason, lipid bilayer coatings and other biomimetic membrane-based strategies have attracted increasing interest as potential alternatives to PEG-based antifouling coatings.

Different methods have been explored to coat silica particles with lipid bilayers. Among them, the vesicle fusion method is the most widely applied [20]. It resembles the adhesion-fusion process used to form supported lipid bilayers (SLB) on planar surfaces [21]. In this method, a solution of vesicles is incubated with silica particles with or without external agitation (e.g., sonication or vortexing). The vesicles first adsorb onto the particle surface, followed by their rupture leading to the formation of a homogeneous lipid bilayer coating on the particle [22,23,24,25]. Another well-studied approach to coating silica particles is the lipid film hydration method, adapted from techniques to prepare liposomes [26]. Here, lipids are dried to form a thin film of lipids, which is then hydrated by adding a colloidal suspension of silica particles, typically followed by vortexing or sonication. During this process, liposomes adhere to the surface of the particles to create a lipid bilayer coating [27,28]. Finally, the on-particle solvent-assisted coating (OPSALC) method, previously explored by our group, offers an alternative approach to coat the surface of particles with lipid bilayers [18]. In this case, lipids and silica particles are mixed in a water miscible solvent under stirring. A buffered aqueous solution is then added to the solution, gradually increasing the fraction of water in the solution, and driving the lipids to assemble on the particles’ surface, resulting in a lipid bilayer coating [18].

**Figure 1:**
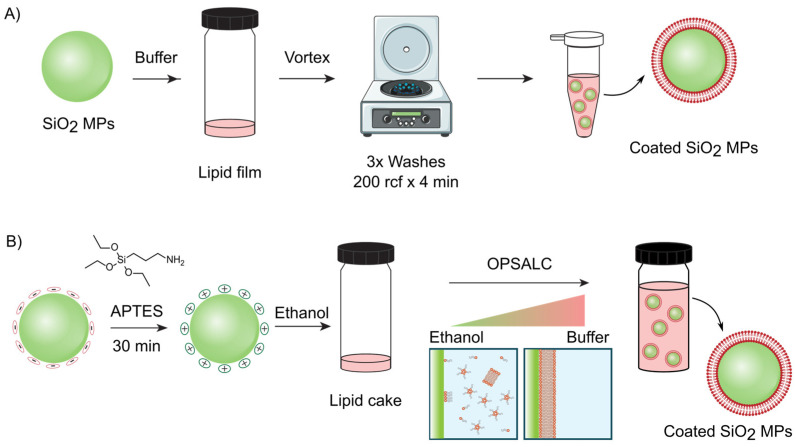
Schematic representation of the coating process of silica MPs through (**A**) the lipid film hydration method and (**B**) the OPSALC method.

**Chart 1:**
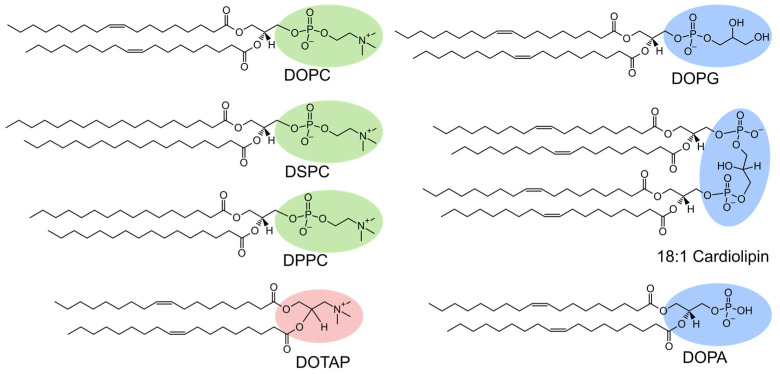
Chemical structures of the lipids used in this study with zwitterionic (green), cationic (red), and anionic (blue) headgroups.

In this study, silica microparticles (MPs) were coated with lipid bilayers using both the lipid film hydration method (Figure 1A) and the OPSALC technique (Figure 1B). The lipid film hydration method was initially applied to native silica microparticles as a simple and widely used coating strategy. When purely anionic lipids could not be assembled due to electrostatic repulsion with the negatively charged silica surface, the OPSALC method, combined with APTES functionalization, was employed to enable electrostatically favorable assembly of DOPG. The OPSALC method allows for using organic solvents, which may be important when particle surfaces do not support liposome rupture. A range of phospholipids (Chart 1) will be studied, varying in their charge and structure, i.e., zwitterionic (phosphatidylcholine, PC) vs. cationic (trimethyl ammonium propane, TAP) and anionic (phosphatidylglycerol, PG or cardiolipin) as well as saturated vs. unsaturated. The coating coverage of the particles will be assessed using fluorescence microscopy, and the protein corona will be evaluated as a function of the different coatings.

Previous studies have demonstrated that coatings and capping molecules with different charges can tune the composition of the protein corona [29,30], which supports the hypothesis that phospholipid charge and membrane phase will have a similar effect on the protein corona formation. This study aims to provide a clearer understanding of how to select a suitable method to coat silica microparticles depending on the aimed-at lipid composition, and how the protein corona is influenced by the mobility and charge of the lipid layer on the silica microparticles. Gaining these insights will support future design and optimization of silica microparticles for biomedical applications.

## 2. Results and Discussion

**Figure 2:**
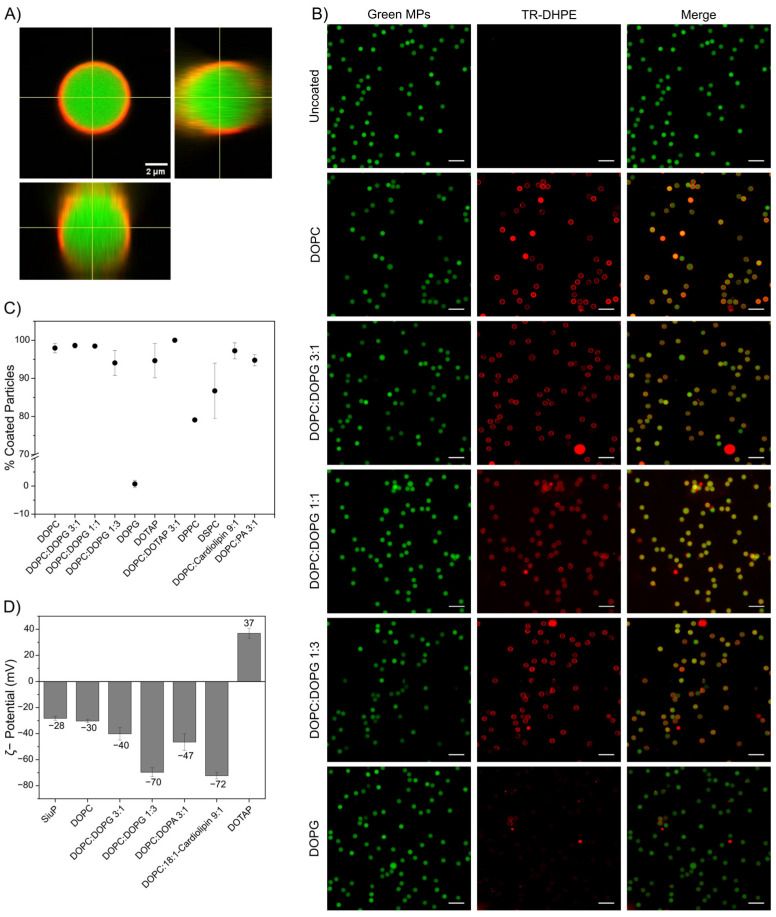
Characterization of the lipid-coated fluorescent SiO_2_ MPs. (**A**) Confocal fluorescence microscopy image and orthogonal views of DOPC-coated fluorescein-stained SiO_2_ MPs prepared following the lipid film hydration method. The lipid mixture contained 0.1% of TR-DHPE. (**B**) Fluorescence microscopy images of lipid-coated SiO_2_ MPs. Scale bars equal 20 µm. Images are enhanced individually for optimal visualization. The lipid mixture contained 0.1% of TR-DHPE. All images were recorded using a Zeiss Confocal microscope except DOPG:DOPC 1:1, which was recorded using an Olympus microscope. (**C**) Percentage of coated particles for each formulation based on fluorescence microscopy images (number of analyzed particles ≥300 for each sample). Data represented as mean ± standard deviation for *n* = 2 independent batches. (**D**) ζ-Potential of the different coated particles in 5 mM NaCl. Data represented as mean ± standard deviation for *n* = 5 measurements.

SiO_2_ MPs were attempted to be coated with different phospholipids of varying headgroups and alkyl chains (Chart 1) following two methods, the lipid film hydration and the OPSALC method. First, DOPC was assembled on the surface of SiO_2_ MPs following the lipid film hydration method (Figure 1A). For visualization of the lipid coating using fluorescence microscopy, a low percentage (0.1 mol%) dye-functionalized lipid (TR-DHPE) was added to the different lipid compositions, while the silica microparticles were functionalized with fluorescein. As evident from the fluorescence micrographs (Figure 2A,B), a homogeneous TR-DHPE emitting shell was detected, while inside the particles, only fluorescein was detected. When counting the number of fully coated microparticles, the data showed that 98% of the microparticles had been fully coated (Figure 2C). These observations confirm that the lipid film hydration method provides a successful protocol for homogeneously coating the surface of SiO_2_ MPs with DOPC. DOPC is in the fluid phase at room temperature (Tm ≈ −20 °C), which promotes vesicle adsorption and rupture on hydrophilic silica surfaces. Vesicle fusion on silica is a well-established route to support lipid bilayer formation. The homogeneous fluorescent shell observed here is, therefore, consistent with the formation of a lipid bilayer coating, although direct measurements of lipid mobility (e.g., FRAP) were outside the scope of this study. Generally, homogeneous coatings are believed to be beneficial for achieving good microparticle biocompatibility and biodistribution. The zeta-potential of DOPC-coated SiO_2_ MPs was -30 mV (Figure 2D), which is comparable to uncoated particles (−28 mV) and in line with previous reports [31]. This minimal shift likely reflects that zeta-potential measures the potential at the slipping plane [32], which is not strongly affected by zwitterionic DOPC. Therefore, the coating produces little change in zeta-potential despite clear formation of the lipid coating observed by microscopy.

Successful lipid coating of silica microparticles was also achieved using DOPC:DOPG lipid mixtures at molar ratios of 3:1, 1:1, and 1:3 when applying the same lipid film hydration method as used for DOPC. For 3:1 and 1:1 DOPC:DOPG lipid mixtures, TR-DHPE fluorescence intensity was uniformly distributed around the particles, with more than 98% of microparticles appearing fully coated (Figure 2B,C), similar to the results obtained with DOPC only. However, at 1:3 DOPC:DOPG, the fraction of fully coated particles dropped to around 90% (Figure 2C).

This reduction may arise from electrostatic repulsion between negatively charged DOPG and the negatively charged silica surface. This is in agreement with the fact that when only DOPG was used, no fluorescent layers, neither partial nor complete, were observed on the SiO_2_ MPs using the lipid film hydration method (Figure 2B,C). Another possible mechanism that could cause the lower DOPG coverage may be compositional asymmetry, causing preferential adsorption of DOPC on the microparticle surface, as has been reported previously [33]. Richter et al. observed that in DOPC:DOPS mixtures, DOPS preferentially adsorbed to planar mica surfaces, leading to unequal distribution of lipids between the two leaflets compared to supported lipid bilayers on planar silica surfaces [34]. The preferential adsorption of DOPC to silica may result in incomplete overall coverage.

**Figure A1:**
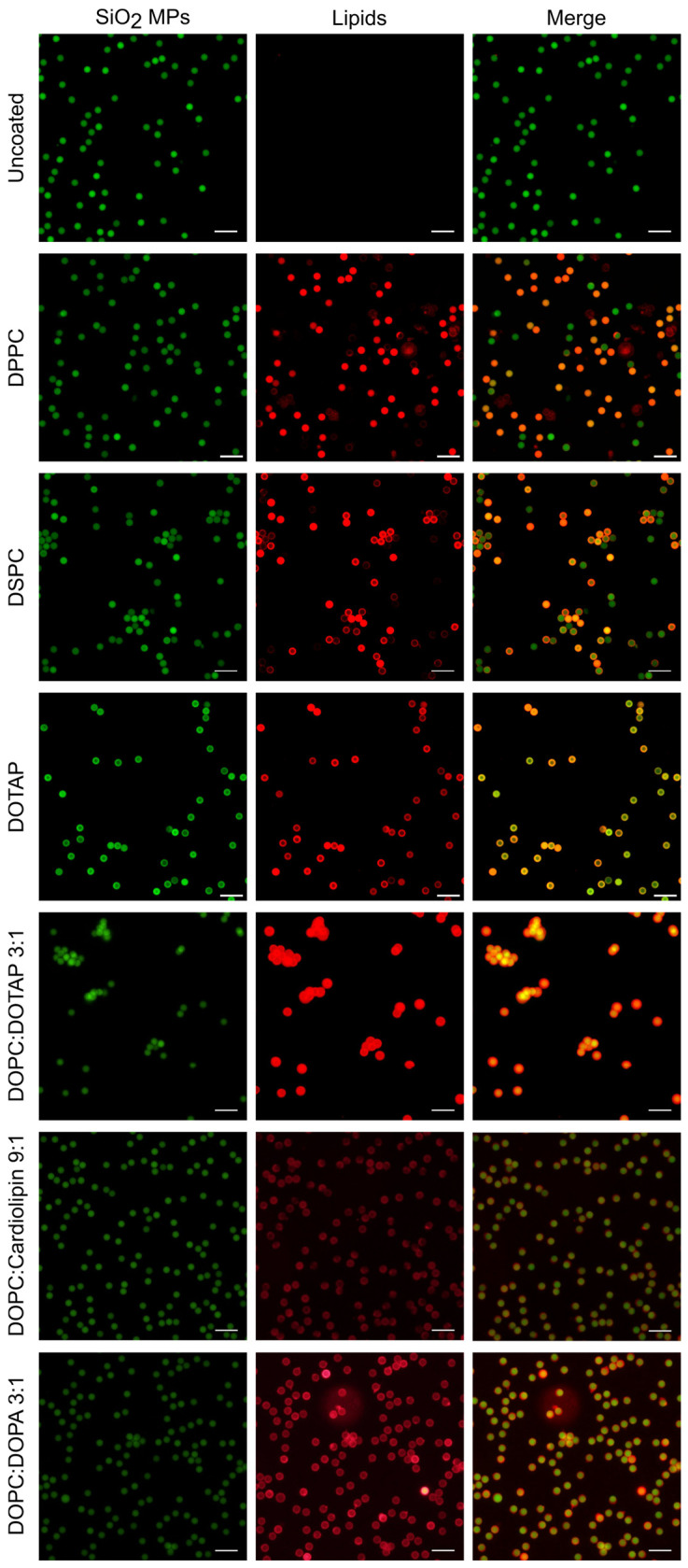
Fluorescence microscopy images of lipid-coated green, fluorescent SiO_2_ MPs. Scale bars equal 20 µm. Uncoated (the same image as in Figure 2B, upper row) and DSPC-coated particles were recorded using a Zeiss Confocal microscope, DPPC and DOTAP-coated particles were recorded using a Nikon A1 Confocal microscope, and DOPG:DOPC 3:1, DOPC:Cardiolipin 9:1, and DOPC:DOPA 3:1 coated particles were recorded using an Olympus microscope.

As expected, increasing the DOPG content led to more negative zeta-potential: from −30 mV for DOPC-only coatings to −40 mV at 3:1 (25 mol% DOPG), and further to −70 mV at 1:3 (75 mol% DOPG) coatings (Figure 2D). A similarly negative surface charge of −72 mV was observed when cardiolipin was used in a mixture with DOPC in a 1:9 ratio, indicating that the doubly negatively charged cardiolipin is present in the lipid coating on the SiO_2_ MPs. Fluorescence images show a high fraction (97%) of uniformly coated particles applying the hydration method (Figure A1). Incorporating cardiolipin in the coating can overcome the limitations of DOPG when more negatively charged surfaces are required. Furthermore, when DOPA was used in a mixture with DOPC at a 1:3 ratio (25 mol% DOPA), the zeta-potential was −47 mV, similar to that of 25 mol% DOPG (−40 mV); however, at a slightly lower percentage of coated particles (95%, Figure 2C,D). Twenty-five mol% DOPA-coated particles presented a clear red, fluorescent layer around the microparticles, clearly indicating that DOPA is another candidate of a negatively charged lipid that can be successfully used to coat SiO_2_ MPs (Figure A1). Overall, different negatively charged phospholipids can be incorporated in DOPC coatings to obtain lipid-coated SiO_2_ MPs in a range of negative zeta-potentials.

**Figure 3:**
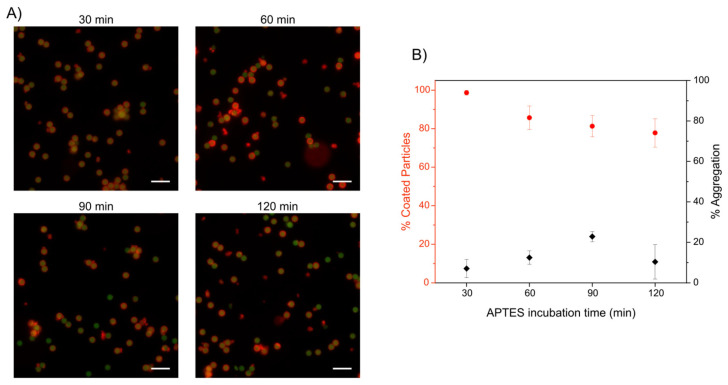
(**A**) Fluorescence microscopy images of green, fluorescent SiO_2_ MPs coated with 0.1% of TR-DHPE containing DOPG following the OPSALC method after APTES silanization for different incubation times. Scale bars equal 20 µm. (**B**) Percentage of coated particles and percentage of aggregated particles for each formulation based on fluorescence microscopy images. Data represented as mean ± standard deviation for *n* = 2 independent batches (number of analyzed particles ≥ 300 for each sample).

Because purely anionic DOPG did not assemble on the negatively charged SiO_2_ MPs using the lipid film hydration method, the particles were amino functionalized with APTES to reverse the surface charge and enable electrostatic attraction prior to coating via the OPSALC method (Figure 1B). This approach was chosen to enable the assembly of purely anionic lipid coatings, rather than to directly compare the coating efficiencies of the two methods under identical surface chemistry conditions. Amino-functionalization with APTES was carried out for 30, 60, 90, or 120 min to create a positively charged surface (+40 mV zeta-potential), promoting electrostatic interactions with the DOPG. To this end, the amino-functionalized particles were coated with DOPG via the OPSALC [18]. Using this approach, 98% of the obtained particles exhibited a full DOPG coating for 30 min of silanization. The DOPG coating is visible as a homogeneous red, fluorescent layer around the green particles in Figure 3. Surprisingly, despite the high coating efficiency, DOPG-coated particles aggregated more than DOPC:DOPG-coated microparticles, which is counterintuitive due to their higher surface charge. Increasing the amino-functionalization time up to 120 min reduced the apparent fraction of fully coated particles to 80%, increased aggregation, and produced less homogeneous coatings with protrusions (Figure 3A,B). These observations may be explained by defects in the lipid coating of the particles, possibly in the nanometer range, leading to aggregation of particles with patches of opposite charge. This is supported by the fact that a higher expected degree of silanization, with potentially rougher surfaces, increased aggregation. Overall, the shortest incubation time of 30 min yielded the highest fraction of fully coated particles (98%) with minimal aggregation.

In contrast, coating silica microparticles with the cationic lipid DOTAP using the lipid film hydration method resulted in a high fraction (95%) of coated particles (Figure 2C). Interestingly, DOTAP particles did not exhibit a narrow red, fluorescent layer around the particles, but instead, they showed a much wider yellow, fluorescent ring, which seemed to partially penetrate the particle, and the green fluorescence of the particles displayed a ring rather than a full sphere, as it does for all the other coated particles (Figure A1 and Figure 2). Due to the positive charge of DOTAP and relatively small size, it is conceivable that the lipids partially penetrate the particles, resulting in the observed thicker layer and yellow color caused by the colocalization of the green and red fluorescence.

The zeta-potential of DOTAP-coated SiO_2_ MPs (+37 mV) was consistent with the cationic nature of DOTAP and indicated a positively charged surface (Figure 2D). When 25 mol% DOTAP was mixed with DOPC, all particles were coated, but aggregated immediately (Figure A1). This suggests that when DOTAP was introduced into a DOPC coating, the particles’ surface charge was neutralized from the −30 mV of DOPC-only-coated particles, as expected.

All lipids mentioned above form bilayers that are in the liquid phase at room temperature, while DPPC and DSPC are expected to be in the solid phase [35], and, therefore, lipid film hydration for these two lipids was performed at 10 °C above each lipid’s chain melting temperature in the fluid phase. Fluorescence microscopy revealed that the majority of the particles that were coated with DPPC lipids exhibited red fluorescence intensity across the entire particle, indicating full penetration of the lipids into the particles. In contrast, when coating the silica particles with DSPC lipids, the majority of the particles exhibited a red, fluorescent shell around the particles, indicating that the particles were coated without observing lipid penetration (Figure A1). The higher thermal energy during the coating process, in combination with the smaller footprint of DPPC (Chart 1) compared to DOPC and the shorter chain length of DSPC, might cause the increased penetration of the DPPC lipids into the particles. Additionally, the fraction of fully coated particles was 85% for DSPC and 79% for DPPC (Figure 2C), which is lower than that observed for DOPC (98%) coated particles.

Partial coating has also been reported for DPPC bilayer formation on planar surfaces [36,37], where continuous SLBs required heating above 60 °C (more than 10 °C above the chain melting temperature), whereas room-temperature deposition produced patchy or incomplete layers. We hypothesize that a similar effect occurs on microparticles, where the assembly of saturated lipids during lipid film hydration shows the lowest homogeneity between the microparticles compared to all other coatings formed via the film hydration method. Optimizing the coating protocol, at sufficiently high temperatures combined with a controlled cooling rate, could improve the uniformity of DPPC and DSPC coatings on silica microparticles.

Taken together, these results demonstrate that SiO_2_ MPs can be reproducibly coated with phospholipids of varying charge and alkyl chain composition, although the optimal coating method must be adapted depending on the specific formulation. The film hydration method is mainly used for phosphatidylcholine-based formulations, whereas OPSALC is employed for fully anionic coatings on cationic pre-functionalized particles. Having established robust and versatile coating protocols, the next step is to investigate how these different coating formulations influence protein corona formation.

**Figure 4:**
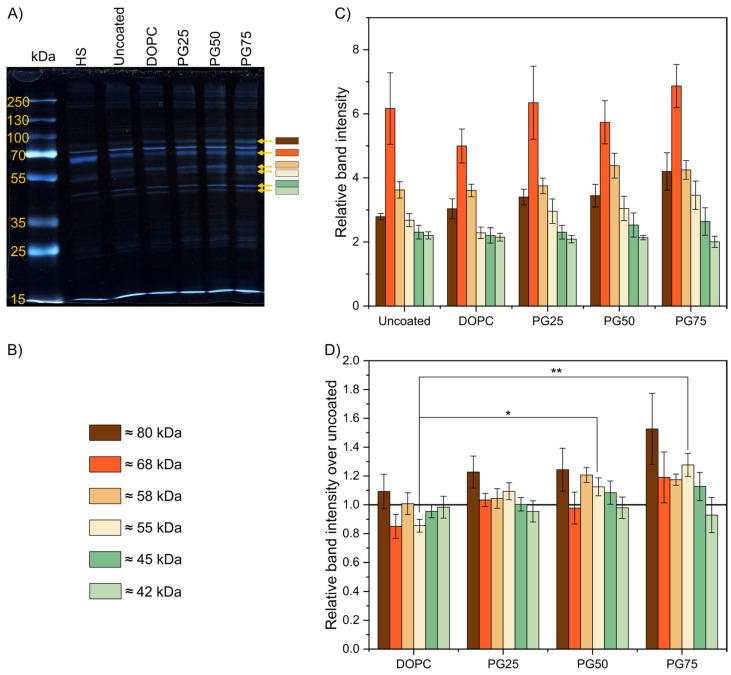
Protein corona quantification based on SDS-PAGE. (**A**) Inverted images of SDS-PAGE gels loaded with, from **left** to **right**: molecular weight ladder, HS, uncoated and coated particles with DOPC, DOPC:DOPG 3:1 (PG25), DOPC:DOPG 1:1 (PG50), DOPC:DOPG 1:3 (PG75). (**B**) Protein molecular mass legend. (**C**) Relative intensity of the band normalized with respect to the total integrated intensity of the lane. Represented as mean ± SE of *n* = 4 gels. (**D**) Relative band intensity normalized to the uncoated SiO_2_ MP intensity. Represented as mean ± SE of *n* = 4 gels. Statistical analysis made with one-way ANOVA with post hoc Tukey test. * indicates 0.05 significance and ** indicates 0.01 significance.

**Figure A2:**
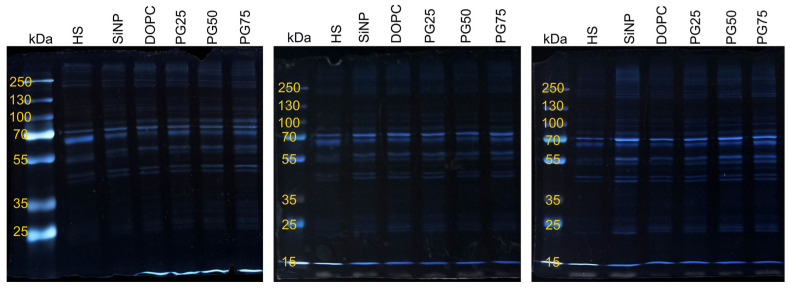
Inverted images of SDS-PAGE gels loaded with, from **left** to **right**: molecular weight ladder, HS, uncoated, DOPC-, DOPC:DOPG 3:1 (PG25)-, DOPC:DOPG 1:1 (PG50)-, DOPC:DOPG 1:3 (PG75)-coated particles.

To this end, silica microparticles coated with DOPC or DOPC:DOPG mixtures at varying molar ratios were incubated with 60% (*v*/*v*) human serum (HS) diluted in DPBS for 20 min at 37 °C. This relatively high serum concentration was chosen to approximate physiological protein levels and to promote competitive adsorption of serum proteins onto the particle surface. The corona-covered microparticles were washed by repeated centrifugation and resuspension; subsequently, the adhered proteins were detached and analyzed via SDS-PAGE (Figure 4A,B and Figure A2). Most of the proteins detected on the microparticles were also present in the control (serum processed without particles, HS), likely residual proteins remaining adhered to the tube walls, despite repeated washing. The similarity in band patterns and intensities between uncoated and lipid-coated particles makes the visual inspection of the formulations difficult.

Densitometric analysis, quantifying absorption of light of each of the bands of the gels, provided a more detailed evaluation (Figure 4C,D). Overall, all samples showed the same 6 bands of proteins varying from 42 kDa to 80 kDa, albeit in different ratios. Coated SiO_2_ MPs exhibited distinct protein band patterns compared to uncoated particles, which varied between different coatings. DOPC-coated particles showed the lowest overall protein adsorption, consistent with the intrinsic antifouling properties of zwitterionic DOPC [38]. Incorporating increasing amounts of DOPG increased protein adsorption proportionally, with 75 mol% DOPG producing the highest level of protein adsorption. The most notable and only significant changes occurred in the 55 kDa band, which was minimal for DOPC and increased with increasing DOPG content, with a 75% increase from DOPC-only to 75 mol% DOPG. Additional variations were observed in the 42 kDa and 45 kDa bands: the 45 kDa band followed a similar trend to the 55 kDa (it increased with increasing DOPG), while the 42 kDa band decreased with increasing DOPG. Despite the small absolute changes in the amounts of adsorbed proteins, clearly, small changes in the coating formulation already produce significant changes in protein adsorption, influencing the composition of the resulting protein corona. In the future, proteomic analysis will be performed to identify the predominant proteins responsible for these differences.

## 3. Materials and Methods

### 3.1. Materials

1,2-Dioleoyl-sn-glycero-3-phosphocholine (DOPC), 1,2-dipalmitoyl-sn-glycero-3-phosphocholine (DPPC), 1,2-distearoyl-sn-glycero-3-phosphocholine (DSPC), 1,2-dioleoyl-sn-glycero-3-phospho-(1′-rac-glycerol) (DOPG), 1,2-dioleoyl-sn-glycero-3-phosphate (DOPA), 1′,3′-bis [1,2-dioleoyl-sn-glycero-3-phospho]-glycerol (Cardiolipin), and 1,2-dioleoyl-3-trimethylammonium-propane (DOTAP) were purchased from Avanti Polar Lipids (Alabaster, AL, USA). DiagNano green, fluorescent silica microparticles (SiO_2_ MPs, diameter 5 μm, DNG-L034), with hydroxyl surface functionalization [39] were purchased from CD Bioparticles (New York, NY, USA). Texas Red 1,2-dihexadecanoyl-sn-glycero-3-phosphoethanolamine (TR-DHPE), sodium carbonate anhydrous (99.5%), silver nitrate, and PageRuler Plus Prestained Protein Ladder 10 to 250 kDa were purchased from Thermo Fisher Scientific (Waltham, MA, USA). Sodium chloride (NaCl), 2-[4-(2-Hydroxyethyl)piperazin-1-yl]ethanesulfonic acid (HEPES), calcium chloride dihydrate (CaCl_2_), sodium dodecyl sulfate (SDS), sodium thiolsulfate pentahydrate (99.5%), N,N,N′,N′-tetramethylethylenediamine, formaldehyde solution (37% wt. in water), and methanol (MeOH) were purchased from Sigma Aldrich (St. Louis, MO, USA). Ethanol (99%) and acetic acid glacial were purchased from Fisher Scientific (Landsmeer, The Netherlands). Tris(hydroxymethyl)aminomethane (TRIS) and Dulbecco’s phosphate-buffered saline (DPBS) without calcium and magnesium ions were purchased from VWR (Avantor Science Central, Amsterdam, The Netherlands). Thirty percent Acrylamide/bis solution, 37.5:1, was purchased from BIO-RAD (Hercules, CA, USA). VACUETTE blood collection tube 8 mL CAT Serum Separator Clot Activator red cap-yellow ring were purchased from Greiner BIO-ONE (NL) (Kremsmünster, Austria). The water used for all experiments described below was Milli-Q water (MERCK MILLI-Q, Molsheim, France, EQ 7000, ρ > 18 MΩ·cm).

### 3.2. Methods

The zeta potential of SiO_2_ MPs was measured on a Zetasizer Lab-Red, Malvern Panalytical (Malvern, UK), in fivefold in 5 mM NaCl. SiO_2_ MPs were imaged using a Zeiss LSM 880 confocal fluorescence microscope (Oberkochen, Germany). For the green dye, an excitation wavelength of 488 nm and an emission range of 493 nm to 573 nm were used, while for the red dye, an excitation wavelength of 561 nm and an emission range of 580 nm to 689 nm were used. The laser power, gain, and acquisition speed were kept constant across all images. A 20× objective with NA = 0.8 and WD = 0.55 mm was used for the imaging of the coating. All image analysis was performed using Fiji software Image J2.16.0/1.54p (https://imagej.net/software/fiji/downloads, accessed on 11 March 2026) [40]. Z-stacks of images were acquired with a 63× water immersion objective with NA = 1.2 and WD = 0.28 mm. Uncoated and lipid-coated SiO_2_ MPs were imaged using a Nikon A1 confocal laser scanning fluorescence microscope (Shinagawa City, Japan) with a 20× objective. SiO_2_ MPs were also imaged using an Olympus IX71 fluorescence microscope (Tokyo, Japan) with a 20× objective. For the green dye, an excitation filter of a wavelength range of 460 nm to 490 nm and an emission filter of 525 nm were used, while for the red dye, an excitation filter of a wavelength range of 510 nm to 550 nm and an emission filter of a wavelength of 590 nm were applied. The intensity and exposure time were kept constant across all images. Fiji software was used for image and particle analysis.

#### 3.2.1. Coating Silica Microparticles Using the Lipid Film Hydration Method

Firstly, the appropriate amounts of the desired lipids were pipetted from a chloroform stock solution to achieve a total of 0.1 mg lipids. For fluorescent coatings, a 0.1 mol% of TR-DHPE to total lipids was added. The mixture was dried under a gentle nitrogen stream to create a thin film while rotating the vial. The film was placed under vacuum for 1 h to completely remove any residual solvent. Meanwhile, a 1 mg/mL suspension of green, fluorescent silica microparticles (SiO_2_ MPs) in HEPES buffer (5 mM HEPES, 75 mM NaCl, 25 mM CaCl_2_, pH 7.4) was prepared. Next, 100 µL of the SiO_2_ MP suspension was added to the lipid cake and vortexed briefly. Then, it was filled up to 1 mL with HEPES buffer and intermittently vortexed for a few seconds over the course of 1 h. The microparticle suspension was diluted 1:1 with water in an Eppendorf tube and centrifuged at 200 rcf for 4 min. Subsequently, 80% *v*/*v* of the supernatant was removed, and the pellet was resuspended in the same volume of water by vortexing. This washing procedure was repeated 3 times, and the final suspension was adjusted to the initial volume (Figure 1A). The particles were stored at 4 °C.

#### 3.2.2. Coating Silica Microparticles Using the OPSALC Method

Green, fluorescent SiO_2_ microparticles (10 mg/mL suspension in ethanol) were added to a solution of APTES (5% *v*/*v*, ethanol). After stirring this mixture for 30 min, the solution was centrifuged three times (100 rcf, 90 s) and redispersed in ethanol. Subsequently, the silanized particles were coated with lipid bilayers following the previously published on-particle solvent-assisted lipid coating (OPSALC) method [18]. Briefly, the appropriate amount of the desired lipids was pipetted from the chloroform stock solution to achieve a total of 40 µg lipids. The solvent was evaporated in a stream of nitrogen, and the film was then kept under vacuum for 1 h to completely remove all residual solvent. The lipids were then redispersed in 160 µL ethanol, and 40 µL of the silanized SiO_2_ MPs (10 mg/mL, ethanol) were added. The solution was stirred at 180 rpm, while 1.8 mL of buffer (10 mM HEPES, 150 mM NaCl, 50 mM CaCl_2_, pH 7.4) was added with a syringe pump at 0.6 mL/min. The resulting suspension was then kept under stirring for 45 min at 50 °C and left to cool down to room temperature. Subsequently, the particles were washed four times by centrifugation at 100 rcf for 2 min with water (dilution 1:1) to remove excess lipids and ethanol (Figure 1B). The particles were stored at 4 °C.

#### 3.2.3. Protein Corona Study

Human whole blood was collected from healthy volunteers at the Blood Donor Service of the Technical Medical Center of University Twente and used within 4 h of withdrawal. The research did not fall within the scope of the Dutch Medical Research Involving Human Subjects Act. Prior informed consent was obtained from all anonymous volunteers, and the blood collection procedure used was approved by the Medical Research Ethics Committee (METC Twente, reference K11-23, date: 8 August 2012). The blood was collected in Vacuette Serum Separator Clot Activator tubes to separate the serum from the blood. Lipid-coated and uncoated SiO_2_ MPs (final concentration of 80 µg/mL) were incubated with 60% (*v*/*v*) human serum (HS) diluted in DPBS from healthy donors at 37 °C for 20 min. The particles were then washed three times by centrifugation at 10,000 rcf for 30 min at 4 °C using cold DPBS. After washing, the supernatant was carefully removed to not disturb the pellet, to which 15 µL of reducing loading buffer was added. As a final preparation, the resulting samples were heated to 98 °C for 5 min prior to the SDS-polyacrylamide gel electrophoresis (SDS-PAGE). The resulting gel was stained with silver nitrate to visualize protein bands. To this end, the gel was fixed in 50% MeOH with 10% acetic acid for 30 min in the 3D orbital shaker. Then, it was washed with 5% MeOH with 1% acetic acid for 15 min in the 3D orbital shaker, followed by three washes with water, 5 min each. For the sensitization, the gel was incubated with a solution of 0.2 g/L of sodium thiosulfate for 90 s and washed three times with water for 30 s. Then, the gel was incubated with 0.2 g/100 mL silver nitrate solution for 30 min while shaking. To develop the gel, the revealing solution was prepared by mixing 3 g of sodium carbonate, 25 µL of formaldehyde, and 1 mL of 0.2 g/L of sodium thiosulfate to give a final volume of 50 mL of water (per gel). After discarding the silver nitrate solution, the revealing solution was added, and the gel was gently agitated until protein bands became visible. This reaction was stopped by incubating the gel in 6% acetic acid for 5 min, after which it was stored in water for imaging.

## 4. Conclusions

In this study, we explored various lipid bilayer compositions for coating silica microparticles using different methodologies. The lipid film hydration method allowed coating with a broad range of lipids, favoring fluid lipids at room temperature. Electrostatic interactions were a key determinant: purely anionic coatings were not achievable on the naturally negatively charged particles, while cationic coatings, though attainable, showed partial internalization into the particles, probably due to strong ionic interaction. Incorporating varying amounts of anionic phospholipids into DOPC enabled the formation of negatively charged coatings across a range of compositions. Fully anionic coatings could only be successfully obtained using the OPSALC method with a pre-silanization step to achieve charge reversal for improved electrostatic attraction of the lipids. A number of these lipid-coated particles with a variety of coating compositions were brought into contact with human serum, and the resulting protein corona was analyzed via SDS-PAGE. It revealed that the lipid composition effectively modulates the profile of adsorbed proteins. While the method is sensitive to experimental conditions, it provides a useful approach for comparing initial protein adsorption on coated particles. For future studies, nano-sized systems are preferred, as their higher surface-to-volume ratio amplifies the influence of coatings on protein corona formation [6]. Further investigations, combining controlled lipid compositions with proteomic analysis, are needed to fully exploit the tunability of lipid bilayers for tailoring particle surfaces to specific biomedical applications.

## Data Availability

Data are contained within the article.
